# Design of EEG based thought identification system using EMD & deep neural network

**DOI:** 10.1038/s41598-024-64961-1

**Published:** 2024-11-04

**Authors:** Rahul Agrawal, Chetan Dhule, Garima Shukla, Sofia Singh, Urvashi Agrawal, Najah Alsubaie, Mohammed S. Alqahtani, Mohamed Abbas, Ben Othman Soufiene

**Affiliations:** 1 Department of Data Science, IoT, Cybersecurity (DIC), G H Raisoni College of Engineering, Nagpur, Maharashtra India; 2 Department of Computer Science Engineering, Amity School of Engineering & Technology, Amity University, Maharashtra, India; 3 Department of AI, Amity School of Engineering & Technology, Amity University, Noida, India; 4 Department of Electronics & Telecommunication Engineering, Jhulelal Institute of Technology, Nagpur, India; 5 Department of Computer Sciences, College of Computer and Information Sciences, Princess Nourah bint Abdulrahman University, P.O. Box 84428, 11671 Riyadh, Saudi Arabia; 6 Radiological Sciences Department, College of Applied Medical Sciences, King Khalid University, 61421 Abha, Saudi Arabia; 7 Space Research Centre, BioImaging Unit, University of Leicester, Michael Atiyah Building, Leicester, LE1 7RH UK; 8 Electrical Engineering Department, College of Engineering, King Khalid University, 61421 Abha, Saudi Arabia; 9 PRINCE Laboratory Research, ISITcom, Hammam Sousse, University of Sousse, Sousse, Tunisia

**Keywords:** Brain computer interface (BCI), Electroencephalography (EEG), Central nervous system (CNS), Empirical Mode decomposition (EMD), Deep Neural network (DNN) etc, Cardiology, Health care, Energy science and technology

## Abstract

Biological communication system for neurological disorder patients is similar to the Brain Computer Interface in a way that it facilitates the connection to the outside world in real time. The interdisciplinary field of Electroencephalogram based message depiction is gaining importance as it assists the paralysed person to communicate. In the proposed method a novel approach of feature extraction is done by Empirical Mode Decomposition on non- stationary & non-linear kind of EEG signal. EMD helps in the effective time frequency analysis by disintegrating the EEG signal in the form of six Intrinsic Mode Functions with help of the frequency components. In all nine features are extracted from the decomposed IMFs so as to predict the states or messages of the patient. The above computed features are then served to the Deep Neural Network to perform the classification. The performance of suggested method is studied through applying it to the acquired database generated by the designed hardware as well as also in real time message depiction. The maximum classification accuracy 97% for the acquired database & 85% in real time are obtained respectively by comparative analysis. The command messages generated from the proposed system helps the person suffering from neurological disorder to establish the communication link with the outside world in an efficient way. Thus, the proposed novel method shows better performance in real time message depiction purpose as related to other existing methods.

## Introduction

Human Machine Interface like Brain Computer Interface (BCI) improves drastically the life of people suffering from severe disabilities. BCI helps to record the brain activity signal in two ways viz. invasive and non-invasive approach. In the former electrodes are applied on or within the brain surface while in the later one it is applied on the scalp. So, it is going to help the person to communicate by operating computer programs to control the neuroprosthesis^1^. It is a technology that transforms controlled choices using brain activity signals into certain commands. To initiate the command, brain activity signals namely evoked related potentials (ERP) are used^2^. It also shows the inspiring advancement from the first human evidence. Human Beings suffering from long lasting paralysis are able to express their thoughts with the help of typing at dynamic rates or controlling the robotic arm to perform holding action like drinking^3^.

Brain computer interfaces help to develop a control & communication application by recording the signal from the brain for person whose organs functionality are impaired^4^. It develops a co-operative application which helps to decipher the brain activity signals into certain messages. In neuroergonomics field this technology significantly assists to propose reflexive BCI devices which perform the primary tasks of monitoring the mental state of the patient^5^. It provides the platform for disabled person to interact with the surroundings by controlling external devices with the help of brain activity signals. Invasive & Non-invasive near-infrared spectroscopy, EEG, are the variety of techniques used to initiate the BCI. Rehabilitative BCI is designed to aid the recovery of neural function as well as to assist the paralyzed person suffering from locked-in syndrome or complete locked-in syndrome by external robotic devices to communicate. The clinical efficiency of BCI also serves as a boon for motor rehabilitation of the person suffering from spinal cord injury or severe stroke^6^.

Brain machine interface shows a quick extension of research as it is capable of providing solutions to the person with physical deficiencies. It is a challenging technology as it ensures the impact of link between man and machine. To achieve the perception in the complication of BCI system use we need to validate the assembly of tactics and its social impact on human^7^. It primarily focuses on how much it benefits the people suffering from neuromuscular disorder. The key step for the same is clinical assessment, authentication & distribution showing the effectiveness of BCI in emulating the natural Central Nervous System (CNS) process. To make BCI system more reliable for communication and controlling tasks it became important that CNS should control these brain signals accurately like spinal motor neurons. Thus, the effectiveness of the BCI outputs depends on the association between two controllers viz CNS & BCI. It is another critical & complex challenge in the development of BCI system^8^.

In the literature work, Electroencephalogram (EEG) signals having finite samples with finite window size utilizes short time Fourier transform that symbolize signal into time frequency approaches. It converts them into images to carry out segmentation of images as decomposition of signal is the foremost step in the work related to signal processing. The values obtained from spectrograms of the image are acting features to the CNN model used for the classification. The techniques work well by using variety of weighted K-NN in terms of accuracy and classification of signal^9^.

In this literature author proposes filter bank instigated and O splines based on EEG rhythm for detection of epilepsy. The energy features taken out from band are classified by SVM and KNN to give accuracy to the extent of 94.88% for seizure non seizure Classification^10^. EEG signal helps to provide communication for the handicapped person using Brain computer interface (BCI). The problem with the EEG signals is its sensitive and non-stationary nature. It gets readily affected with the artifacts & noise due to mental & physical state, mood and posture of the person. Here the author proposes an algorithm which makes use of pruning for EEG signal classification. The Support vector machine is used to make a different group of classifiers. Then pruning is performed on the group with the help of new optimization model by variance of convex algorithm^11^.

In the proposed literature deep learning methods called as convolutional neural network are offered for efficient classification of the networks and early diagnosis of schizophrenia (SZ) in neuropsychiatric patients using electroencephalogram (EEG) signals. In view of capturing the dislocated joints in SZ, the amalgamation of various features based on different parameters of network topology were used. This Multi-Domain Connectome Convolutional Neural Network (MDC-CNN) achieved the performance accuracy of 91.69% over single-domain CNNs. The proposed MDC-CNN accurately distinguish between schizophrenia (SZ) from healthy controls (HC). It is thus useful for the evolution of new diagnostic tools for SZ disorders^12^.

Development of Brain Computer Interface highly depends on the accuracy, efficiency and reliability of the algorithm used for the classification. To investigate the non-linear & non-stationary EEG signals a novel approach of empirical wavelet transform is utilized to disintegrate the signal as it reduces the system complexity & execution time. This method implements analysis of Welch power spectral density to perform selection of single mode and Hilbert transform (HT) for extraction of both signal components of IA and IF. The classification is accomplished using Least Squares Support Vector Machine (LSSVM) classifier and Empirical Wavelet Transform (EWT) so 95.2% and 94.6% of classification accuracy are obtained respectively. The results show that mixture of both techniques helps to attain higher success rate, great potential over existing methods for BCI system^13^.

Adding further response to the biomedical problems related to neurological diseases is given to society, by detecting diseases using electroencephalogram signal. Different types of seizures are classified based on the temple university data of corpus. Empirical mode decomposition is the best decomposition method to excerpt different levels of IF. The corresponding IMFs are given to support vector machine classifier for classification. Different SVM kernel can be used to disperse classes of data that cannot be split in a straight line to segregate nonlinear data^14^.

Different noise components such as baseline wander (BW), power line interference (PLI), and white Gaussian noise (WGN), unavoidably contaminate the Surface electromyography (EMG) signals degrading the accuracy, efficiency & robustness of EMG processing for further applications. In the proposed work a novel filter is designed using variational mode decomposition (VMD) to eliminate all noises as most of the filter’s design until now focus on only one category of noise. Here noisy EMG signal is first disintegrated into an ensemble consisting of band limited modes & then each category of noise is removed separately from each sub-band. The various evaluation metrics used in the analysis of denoising performance from simulated & experimental signals includes percentage reduction in correlation coefficient, the improvement in SNRimp & RMSE. The best performance is achieved for removal of noise like WGN or BW using this method over ensemble empirical decomposition method (EEMD). When the SNR is low PLI noise is also effectively reduced. The results show that VMD based method is sensitive to real spikes & removes the noise from resting states. The proposed filter can be used as a preprocessing technique for EMG signals in a variety of applications like EMG decomposition or gesture recognition^15^.

In the proposed work a novel method of EMD is utilized for feature extraction of EEG signals on publicly available dataset. The decomposition of EMD signal as per its frequency components are obtained in the form of intrinsic mode functions (IMF). Apart from pathological EEG signals, Normal EEG signals have different dispersions, symmetries, spectral & temporal centroids. These physiologically relevant features are extracted in the form of spectral centroid, temporal moments of third order, spectral skew & coefficient of variation from the IMFs. The classification of the calculated features is then done with the assistance of SVM classifier. Investigational results show that detection of seizures & identification of epilepsy patients are efficiently obtained using this feature extraction method^16^.

In the proposed work, an amalgam feature extraction method in EMD domain based on Lempel–Ziv Complexity (LZC) and spectrum centroid, complemented by Sequential Backward Selection (SBS) is used for emotion recognition from Electroencephalogram (EEG) signals. The unstable & non-linear EEG signal is decomposed into multi scale components, and a sequence backward selection is used to eliminate the redundant features & to capture the subtle information for improving performance of emotion recognition using DEAP dataset. The dataset is classified into Valance dimension & Arousal dimension by support vector machine followed by k-nearest neighbor algorithm. The comparative analysis of rhythms of three kinds of EEG signal is done on temporal windows of different lengths. In human-robots interaction system multimodal emotional communication can be possible in real time by using the proposed method for emotion recognition. The experimental results achieve highest accuracy with 1^st^ temporal window^17^.

Prognosis may differ between patients suffering from epilepsy depending on various factors like seizure semiology, etc. An accurate EEG patterns analysis by an epileptologist can help in prediction & treatment of prognosis. Such an epileptologist is not available in most of the hospitals. In the proposed work a hybrid machine learning model is designed to distinguish between Benign Epilepsy and Temporal Lobe through Centro-temporal Spikes (BECTS) as both show different age of onset & seizure types. The first step is the creation of feature matrices which is then processed using support vector machine (SVM) technology followed by ensemble learning to classify the result based on the decision trees. The comparative analysis of this particular ML algorithm with others like KNN, logistic regression, ensemble learning based decision tree & SVM is also done.

A novel approach^18^ for identifying motor imagery (MI) tasks that combines EEG source imaging (ESI) and convolutional neural networks (CNNs) is being developed. The ESI method uses weighted minimum norm estimation (WMNE) and boundary element method (BEM) to evaluate EEG forward and inverse issues, individually. Within motor cortex, it systematically organizes ten scouts to identify areas of interest (ROIs). From the time series of these scouts, features are retrieved utilizing a Morlet wavelet method. CNNs are then utilized to classify MI-related activities efficiently.

The correct decryption of brain activity monitored with the help of electroencephalogram (EEG) is critical to build successful and economical brain-computer interface (BCI) systems. Traditionally, EEG signals are classified without regard for the topological link between electrodes. Neuroscience research has progressively focused on brain dynamics network patterns. So, electrodes eucledian structure may not accurately depict interaction of signal. The graph Laplacian of EEG electrodes is produced using overall signals absolute Pearson's matrix. The GCNs-Net learns generalized features by means of graph convolutional layers. The dimensionality reduction is done by next pooling layer while final prediction is done by fully connected softmax layer^19^.

EEG-based brain–computer interface (BCI) development techniques used recently frequently sacrifice reaction speed or recognition accuracy. With amazing sensitivity and accuracy, this study presents a revolutionary deep learning method in view of scalp EEG-based motor imagery (MI) identification. Graph Convolutional Neural Network (GCN) derived topological features structures from full dataset thereby improving decoding performance .Furthermore, attention mechanism with bidirectional long short-term memory (BiLSTM) is utilized for enhancement of precision and responsiveness^20^.

The importance of taking into account the topological links between EEG electrodes for precise EEG information decoding has been brought to light by recent neuroscience research. A novel structure is built on attention-based residual network known as Graph Convolutional Neural Network (GCN) to fill the gap. This network builds a graph built on topological structure of electrodes and uses raw EEG signals to identify human motor intents. Additionally, in raw EEG motor imagery (MI) data, full-attention architecture with deep residual learning has been proposed to alleviate issue of deeper networks deteriorating performance^21^. Table 1 below shows the analysis of survey done on existing methods.

**Table 1:** Summary of Literature Survey.

Sr. No	Disease	Developed/ Communication System	Technique Used (Decomposition and Classifier)	Remark/ Limitation
1	Epilepsy	Classification of Seizure free, Seizure classes & normal	least square support vector machine & K- nearest neighbor, Taylor Fourier filter bank	Only Diagnosis
		Diagnosis of epileptogenic area	Convolutional Neural Network, STFT	Only Diagnosis
		Epilepsy Detection	Empirical Mode decomposition, Hilbert Transform, Support vector machine	Only Diagnosis
		Diagnosis of seizure	TQWT, Ensemble learning	BONN database used
2	Hearing disorder	Emotion & Facial Action Recognized	Hybrid Fuzzy Approach	System work on real time signal
3	Heart disease	Heart disease Identification	Deep Convolutional Neural Network Classifier	Diseases diagnosis by very less parameters and time required to that is large
4	Paralysis	Wheelchair	Band power, radial basis function	Use of Physical movement
		BCI System	Support Vector machine, Power spectral density	Survey presented, system not implemented
		Communication system with the help of eye blink movement	Window platform- neuro sky interface	Only two messages can communicate
		Wearable rehabilitation device	Neural Network & Support vector machine	Issue of portability
		Robotic Upper limb exoskeleton	Fuzzy logic technique	Robotic upper limb helps to control the wheelchair
		Brain controlled wheelchair	Support vector machine, discrete wavelet transform	Right, left, forward, and backward and stop movement of wheelchair is possible
		Movement of Joystick as per Motor imagery classification	Empirical Mode Decomposition & Band Power, Hidden Markov Models	Only two movement of the joystick i.e. right or left is possible
5	Parkinson	Detection of seizure	Leaf weight quantization	Only diagnosis
6	Physically disabled	Orthotic Exoskeleton arm	Convolutional neural Network	Difficult to wear the arm for the patient
		Locking & unlocking of wheelchair	EMG & EEG Detection	Jaw & eye blinking movement are considered
		Motor imagery tasks classification	Deep Convolutional Neural Network Classifier, short time Fourier transform & continuous wavelet transform	System with image processing, right hand and right leg task considered
		Motor imagery tasks classification	Common Space Pattern & Wavelet Packet	Only two types of EEG signal has been classified on database
		Motor imagery tasks classification	Empirical Wavelet Transform, welch power Spectral density & Hilbert transform	Increases the computational load which maximize the time as well as hardware cost
		Motor imagery tasks classification	Novel ensemble pruning, support vector machine	EEG signal may vary due to mood & posture of the patient
		Wheel chair with embedded robotic arm	steady state visual evoked potentials (SSVEP)	Help in providing control to wheelchair
		Voice controlled automated wheelchair	Speech recognition module	Controlling of wheelchair by voice command but not suitable for person who cannot speak
7	Schizophrenia	Diagnosis of Schizophrenia	Multi domain connectome convolutional neural network, Support vector machine	Only Diagnosis
8	Sleep apnea	No system is developed	Particle Swarm Optimization, Hermite Decomposition, Least Square Support Vector Machine	Only Diagnosis of the diseases has been Done
9	Speech disorder	Emotion Recognition	Convolutional Neural Network	Work on Emotion Recognition
		Control & Communication of smart Devices & Mobile applications	Bayesian Network Classifier, Pair-wise Classifier	BCI implementation using EEG
		Emotion Detection	K-nearest neighbor Classifier	Poor Performance due to distance metrics
		Motor imagery tasks classification	MEMD, CSP, Entropy and Walsh Hadamard Transform	Only two types classes for right & left hand has been obtained
		Classification	EEG & EMG in a hybrid BMI	Used for enhancing of classification
10	Others	Motor imagery tasks classification	Higher order spectral or statistical analysis	Only survey has been done on higher order statistics or spectra
		Motor imagery tasks classification	Convolutional neural network, Deep Learning neural network	Optimization of structure & parameters of the network is needed
		Emotion Recognition	EMD & random forest classifier	Only emotion recognized & no messages has been depicted

Extraction of intrinsic mode functions for efficient decomposition of EEG signals followed by deep learning-based classification for improved accuracy and robustness. Empirical Wavelet Transform (EWT) combined with Least squared support vector machine (LSSVM) classifier achieves 95.2% classification accuracy, reduced computational complexity and execution time achieved higher success rate and potential over traditional BCI systems. Amalgamation of Lempel–Ziv Complexity (LZC) and spectrum centroid for feature extraction with Multi-scale component decomposition using EMD, followed by SVM and k-nearest neighbor classification for real-time emotion recognition enhanced accuracy with real-time applicability. Prognosis Prediction is done using Hybrid Machine Learning Model where Feature matrices creation followed by SVM classification and ensemble learning helps in improved prediction accuracy and robustness in distinguishing between epilepsy types. Effective utilization of Empirical Mode Decomposition (EMD) for capturing complex patterns in EEG signals followed by deep learning method like CNN helps in improving classification accuracy. Filter bank based on EEG rhythm utilizes frequency-specific information for improved classification performance followed by SVM and KNN classification to enhanced accuracy in epilepsy detection with focus on frequency-specific features. Implementation of Variational Mode Decomposition (VMD) for noise reduction followed by EMG signal processing enhanced robustness and accuracy in EMG signal analysis by effectively removing noise components not addressed by other methods. The analysis with previous research shows that no one has developed the hardware for depiction of real time message. Also the application of EMD with DNN is the efficient way to carry out our proposed work.

## Design methodology

In the proposed work, combination of common spatial pattern (CSP) & EMD of scalp electroencephalogram was used. The proposed model helps in real time diagnosis to accurately distinguish between different types of epilepsy^22^. EEG are taken for a novel automatic seizure onset detection amongst epilepsy patients. The first step is filtering, pre-processing & time–frequency decomposition of EEG recordings with the help of EMD & wavelet transform. The only feature called variance is extracted by using CSP which helps in reducing the dimension. For classification purpose a group of ten SVM’s is used & after that post-processing is done. Because of which the recognition rate increases & false detection rate reduces. The proposed work shows that CSP spatial filter helps in clinical application of seizure onset detection effectively^23^.

**Figure 1:**
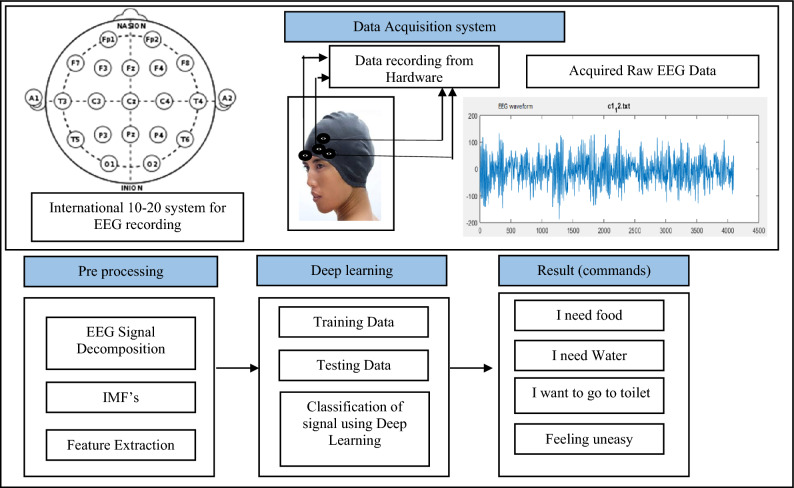
Proposed Architecture using EMD.

The system architecture for the proposed work with detailed description is mentioned in this section. Figure 1 shows design flow for the given system. Here from the original raw database total 216 files of recorded EEG signal are acquired from the hardware. Each file has been built by acquiring the EEG signal for the duration of 10 s. 54 files for each command has been captured so as to get total 216 files for experimental hypothesis. These EEG signal samples are captured with the help of Hardware whose diagram & description is given below. The EEG signal is disintegrated by using EMD which decays it into set of elemental signals called as Intrinsic Mode Function (IMF’s). Non- stationary or non-linear signals like EEG can be very well separated into physically meaningful components of different resolutions using EMD technique making it easier to analyze. In the proposed work we have system structural value various IMF’s function.

In the proposed work machine learning algorithm namely, deep neural network was applied as number of features are more compared to number of samples. Moreover, identification of brain activity is done with the help of extracted features that behave as input to deep neural network classifier. Finally, communicating message can be acquired according to the classified output.

### Institutional review board statement

This study was conducted in accordance with the Declaration of Helsinki and its later amendments or comparable ethical standards and approved by the Institutional Review Board of Department of Data Science, IoT, Cybersecurity (DIC), G H Raisoni College of Engineering, Nagpur, Maharashtra, India under protocol number 630.

### Ethical considerations

Informed consent was obtained from all individual participants included in the study for the publication of identifying information and/or images in an online open-access publication. Copies of the informed consent forms are available upon request. Also written informed consent to participate in the study was obtained from the participants involved.

## EEG experimental hypothesis

**Figure 2:**
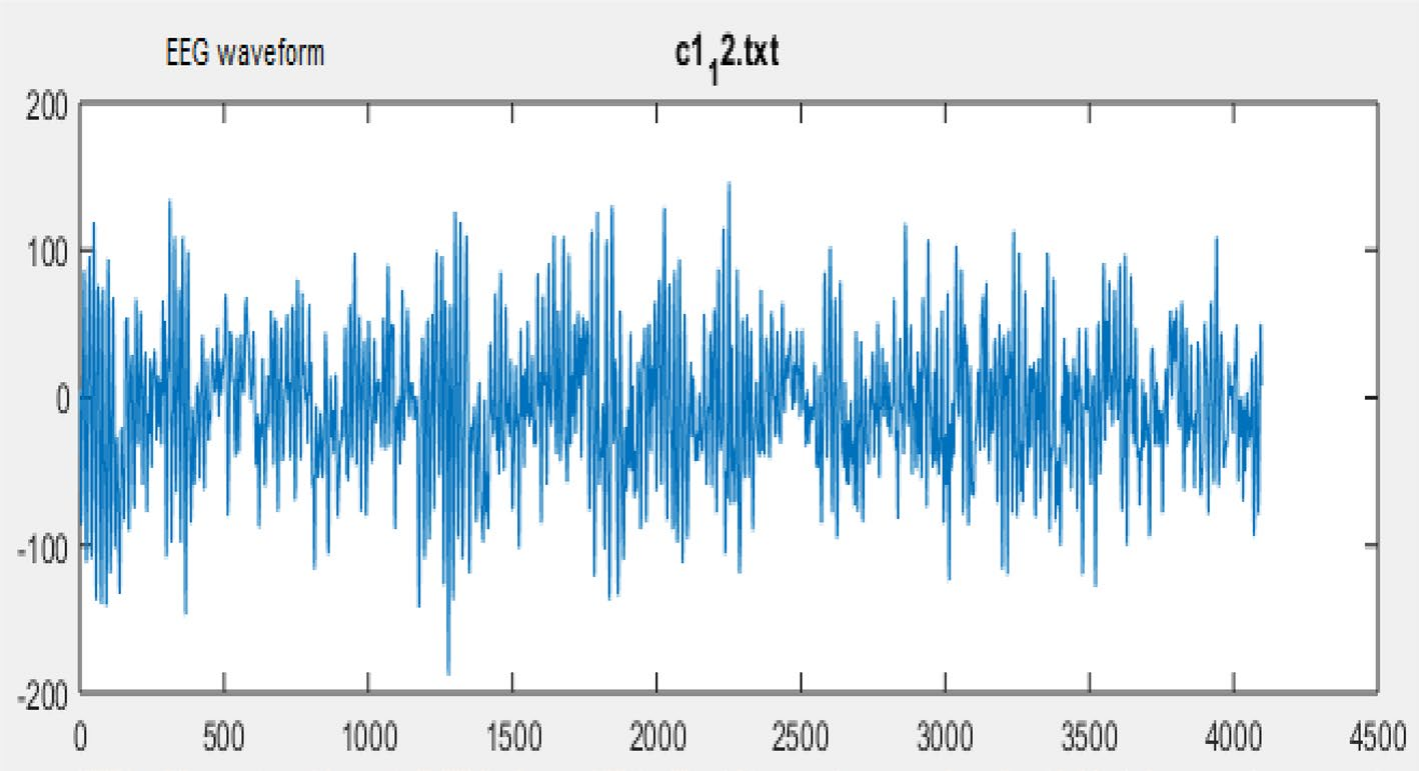
EEG Data Signal Sample waveform.

The person whose sample signal is being taken is called as subject. Figure 2 shows the sample waveform of the EEG signal. During the data collection process the subject are requested to seat on a chair in relax Position. They are then allowed to repeat the sentence without vocalizing and making any type of muscle movement. The four sentences repeated by the subject are nothing but the messages to be delivered by the paralyzed person or the person suffering from neurological diseases.

The protocol is design to classify the four messages for which the signal is acquired during the EEG experimental hypothesis are explained below.

- *I need food*: Subject will continuously think about the message “I need food” mentally for 15 s without any body movement and Vocalization of the message during recording.
- *I need Water:* Subject will continuously think about the message “I need water” mentally for 15 s.
- Without any body movement and Vocalization of the message during recording.
- *Feeling uneasy:* Subject will continuously think about the message “Feeling uneasy” mentally for 15 Seconds without any body movement and Vocalization of the message during recording.
- *I need to go to Toilet:* Subject will continuously think about the message “I need to go to Toilet” mentally for 15 Seconds without any body movement and Vocalization of the message during recording.

**Figure 3:**
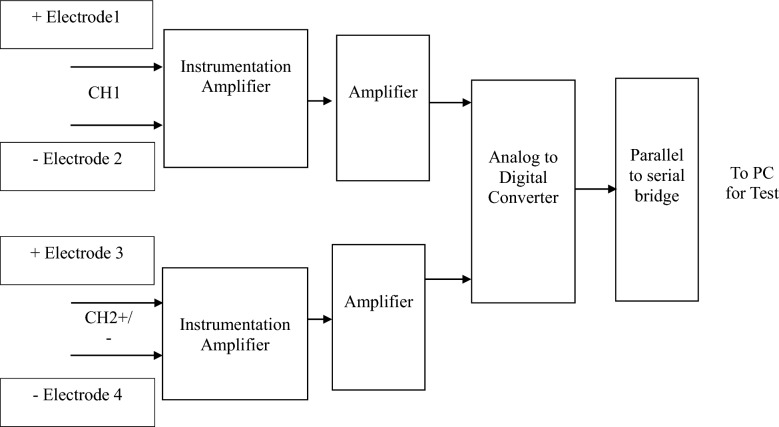
Hardware description diagram for collecting the real time data sample of EEG signal.

Figure 3 shows the hardware description for the proposed work.

The Technical configurations and necessary specifications for each hardware component used in the EEG signal acquisition system are provided below.

- AD683 Op-Amp Summing Amplifier: This component is used to amplify the signals obtained from the EEG electrodes. It's chosen for its low noise, low offset voltage, and other characteristics that make it suitable for amplifying weak bio-potential signals.
- LMC6482 Instrumentation Amplifier: This amplifier further amplifies the signal and helps simplify filter requirements by rejecting line harmonics and wideband interference. Its advantages include low gain drift, low offset drift, and high Common-Mode Rejection Ratio (CMRR).
- ADS1293 Analog to Digital Converter (ADC)**:** This is a 24-bit, 3-channel ADC specifically designed for bio-potential measurements. It converts the amplified analog signals from the EEG electrodes into digital signals for processing. It offers features such as low power consumption, high integration, and various additional functionalities like lead-off detection and SPI interface.
- STM32 Microcontroller: This microcontroller, based on the ARM Cortex-M architecture, serves as the processing unit for the system. It handles communication with peripheral devices and manages data processing tasks. Its low-power characteristics make it suitable for portable applications.

This hardware is basically used to capture the real time EEG signal of the subject which is further described in the following steps.

## EEG signal acquisition

**Figure 4:**
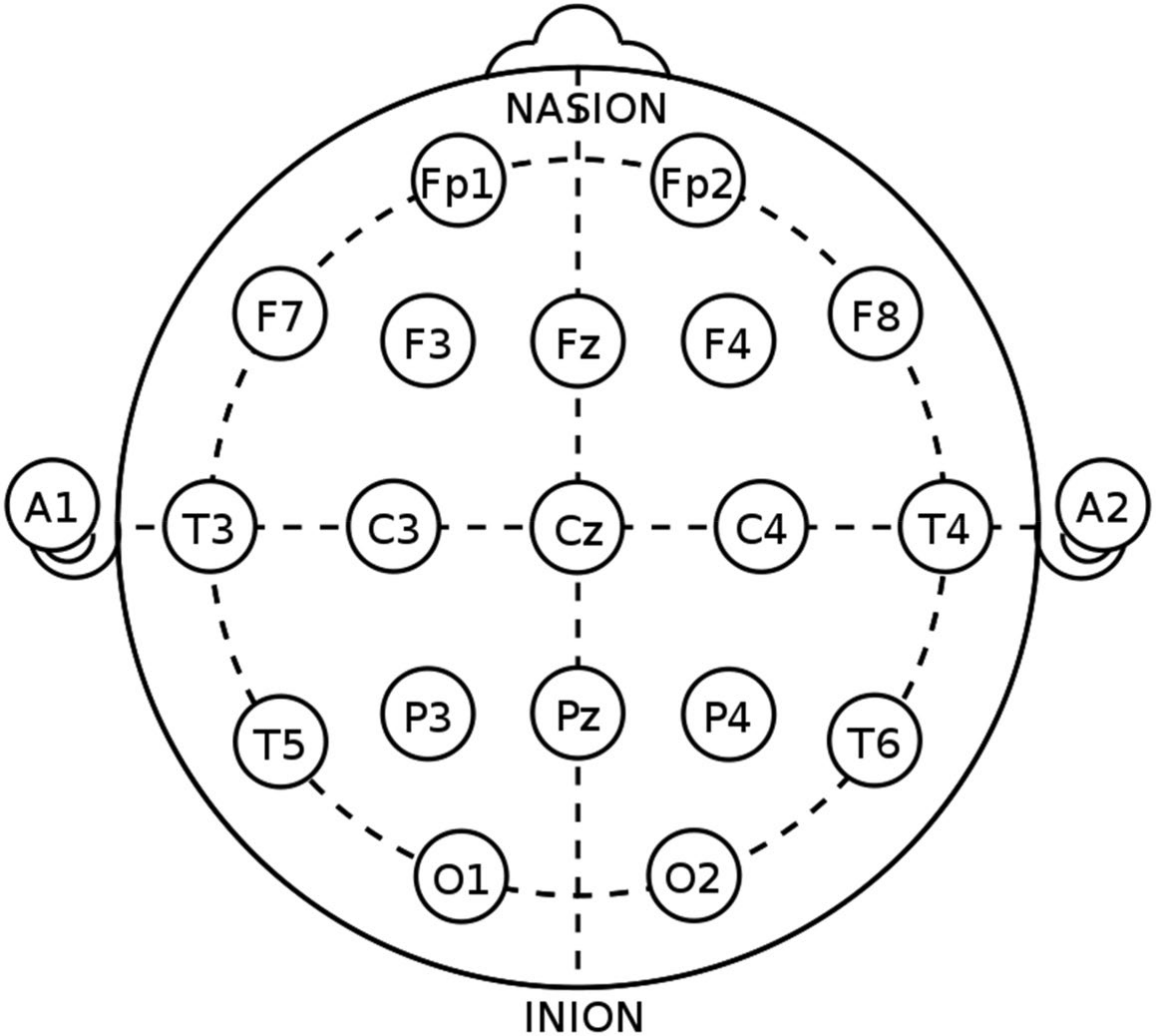
International 10–20 system for EEG recording.

The Human brain is divided into four lobes which is accountable to perform various tasks. Whenever certain work is done the brain will generate the signal regarding the same in the corresponding lobe. The hardware module will acquire EEG signal with five EEG electrode viz. 2 channel and one reference. EEG signal is basically divided into five different frequency bands i.e. from 0.5 to 3.5 Hz Delta, from 3.5 to 7.5 Hz Theta, from 7.5 to 12 Hz Alpha, from 12 and 30 Hz Beta, from 31 Hz and higher Gamma. The electrode is placed on the forehead, about an inch above the point exactly between the two eyebrows. The voltage is measured in µV from the frontal cortex of the forebrain. Such signal can be captured on the surface of the scalp by placing electrode which is nothing but the EEG signal. For this a standard 10–20 system is used which defines the distance to be maintained between the electrodes as shown in the Fig. 4. The useful or considered point in the proposed work are Fp1, Fp2, Fz, Cz and one point below left ear for reference.

**Figure 5:**
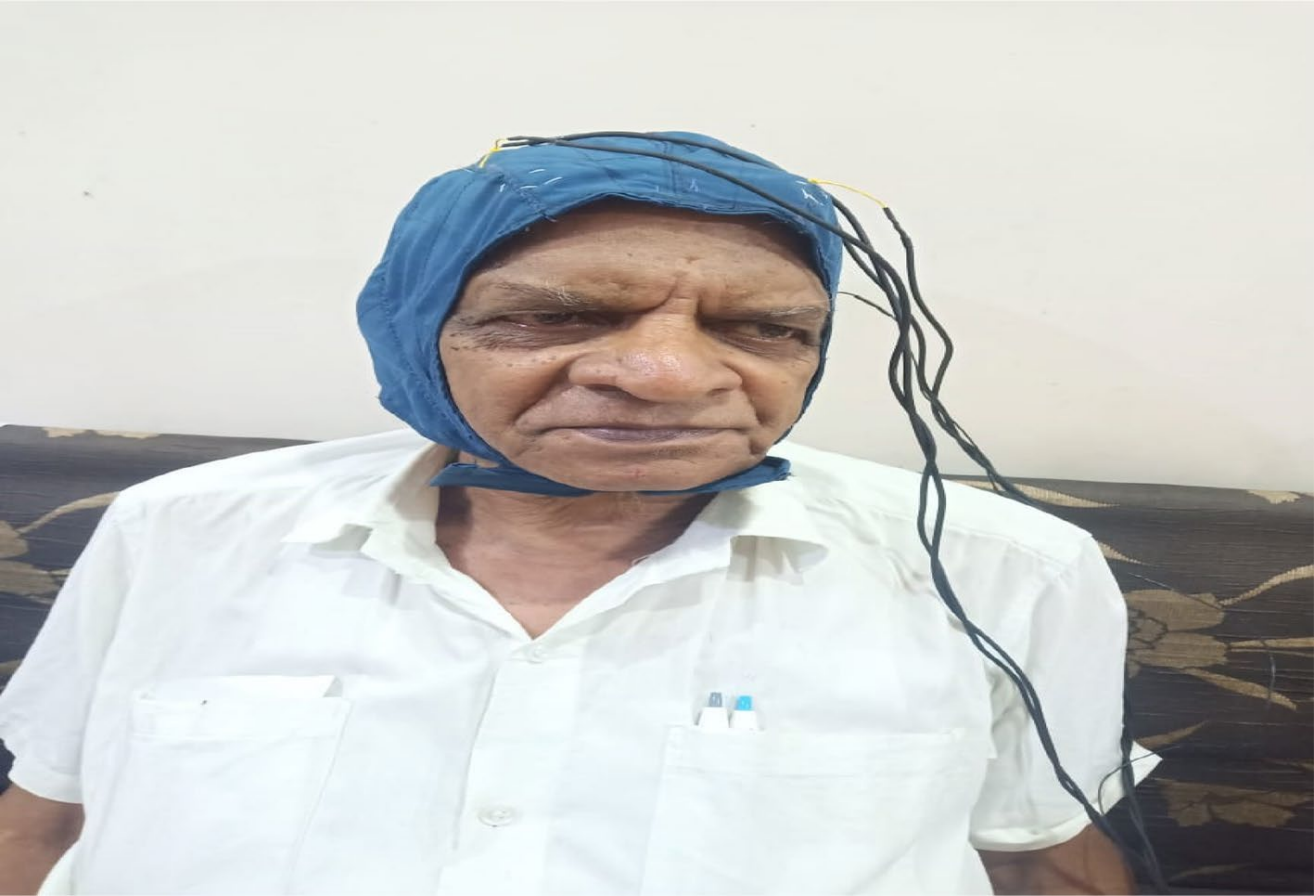
Experimentation using EEG Signal acquisition Hardware for Neurological disorder patients.

Figure 5 shows the experimental setup for acquisition of EEG signal. The EEG signal recording is done between 20 to 128 channels and the sampling frequency which is used to sample the signal is around 250 Hz. EEG Electrode Gold F is used for the same purpose.

**Figure 6:**
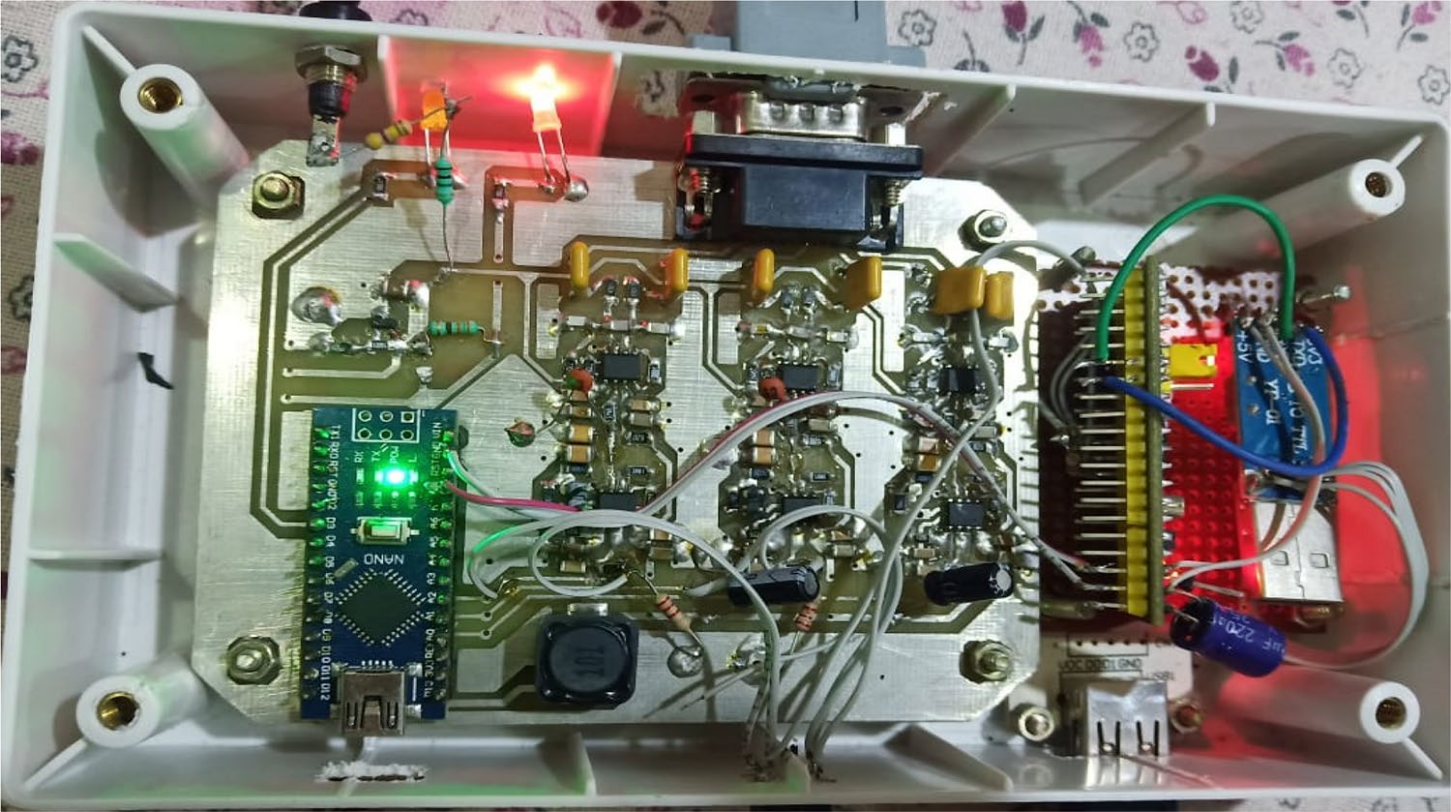
EEG recording device for Data acquisition.

Figure 6 shows the EEG recording device used in the proposed work. Following are the different blocks used in this Hardware and its detailed description one by one.

AD683 is a dual, bipolar, low power op-amp summing amplifier used to amplify the signal obtained from EEG Electrode due to variety of its useful characteristics such as low noise, low offset voltage, high source impedances & unity gain, etc.

LMC6482 is an instrumentation amplifier which is used to further amplify the signal. It basically helps in simplifying the filter requirements as it rejects the line harmonics as well as wide band interference. Low gain drift, low offset drift, low voltage offset, high CMRR & high gain accuracy are some of the advantages of using this instrumentation amplifier.

In the front-end ADS 1293, a 24-bit 3 channel, low power analog to digital converter is used for bio potential measurements. It is used in scalability of medical instruments due to its highly demanding features such as low cost, power & size with exceptional performance & high levels of integration. It increases the performance & power by increasing the bandwidth & sample rate. It also provides different additional features like less input bias current, high CMRR, AC & DC lead off detection, built in oscillator, battery voltage monitoring, standard SPI interface, etc.

STM32 is the ARM Cortex-M 32-bit processor core-based microcontroller. It allows serial & parallel communication with various peripheral devices as well as cameras, motors, sensors & displays, etc. It also has additional feature like RAM & internal flash memory. It is used mostly for portable & low-power applications which even work on small battery.

## Empirical mode decomposition (EMD)

Pre-processing become an important part as the raw EEG signal is not pure & it contains various artifacts & noise. The main causes of noise in EEG signals are EEG equipment, electrodes & lead, electrical interference created due to electrical activity from the eyeball movements, eye blinking, muscle movement & heart. This steps helps in cleaning the EEG signal from artifacts & noise. The filters are used to perform the preprocessing. To remove the drifts & dc components present in the signal high pass filters having cut off frequency of around 1 Hz is employed, as EEG signal is not studied at such high frequencies. After preprocessing the recorded signals is cut in epoch of every few seconds to obtain a large number of features so that statistics can be applied to a single EEG recording.

A data-driven method for time–frequency analysis called Empirical Mode Decomposition (EMD) splits down a signal into Intrinsic Mode Functions (IMFs). Finding local extrema in the signal and building upper and lower envelopes between succeeding extrema are the first steps in the procedure. The first IMF is obtained by subtracting the mean of these envelopes from the original signal, and it meets specific requirements concerning extrema and mean values. The procedure repeats on the residue to extract additional IMFs if these requirements are not satisfied. Until the residue meets a predetermined stopping threshold or turns into a monotonic function, this iterative process is continued. EMD is a useful tool in a variety of signal processing applications due to its adaptive nature and capacity to capture non-linear and non-stationary components.

EEG signals are not linear by nature as well as it is varying which means it contains altered intrinsic modes of oscillation. EEG signal is an amalgamation of variety of signals with various frequency domain values. Hence EMD method is used in the proposed work for breaking down of this complicated signal into sub bands that are small & limited number of components. The decomposed components nearly form the orthogonal basis for the EEG signal. EMD is adaptive & data dependent method to decay the complex EEG signals into numerous oscillatory components. These components are known as intrinsic mode functions. Firstly, the local extrema points were found out for the given EEG signal. Then the data samples are arranged as per their size and median value which is the centre value is calculated from it. Finally individual IMF’s can be calculated with the help of subtraction between median value and input signal. The process of discrimination is then performed to identify that the function can be classified as IMF depending upon two conditions.

**Figure 7:**
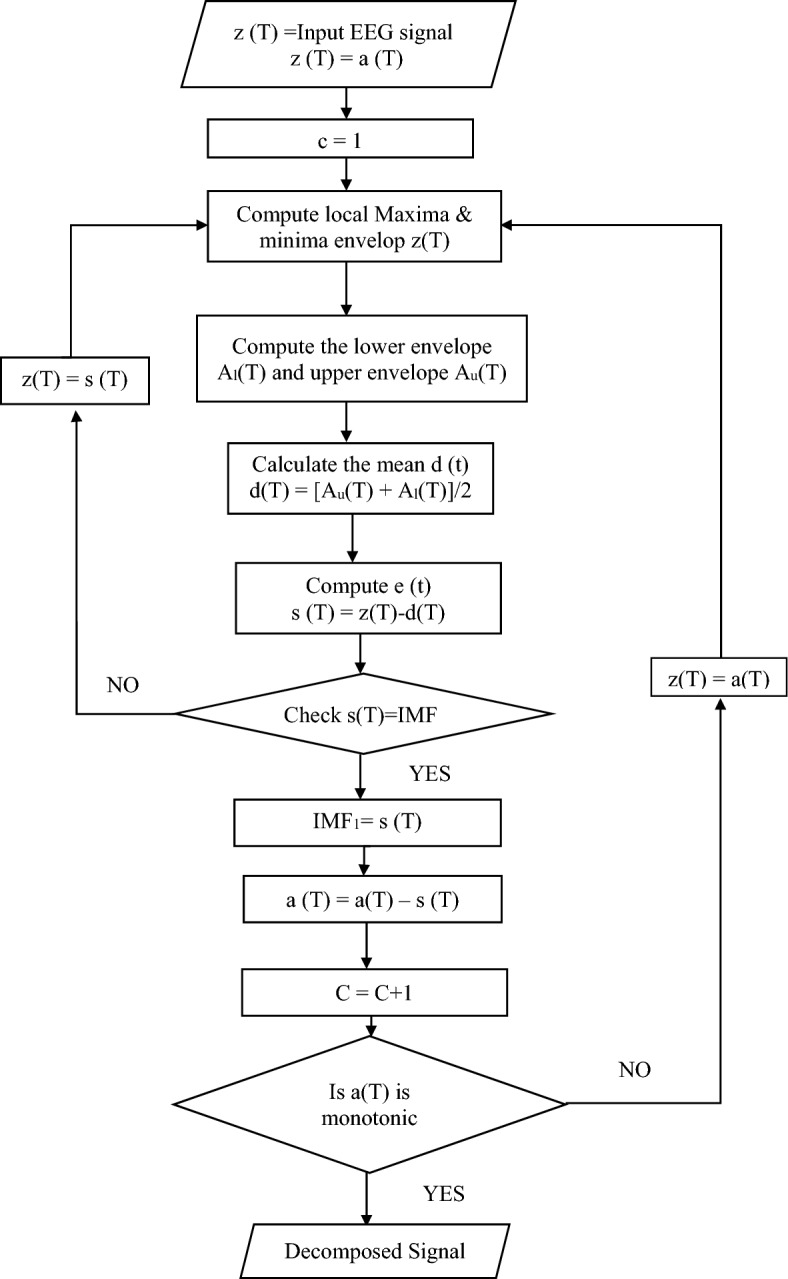
Flowchart for EMD decomposition.

Firstly, in the whole EEG dataset all the zero crossing points as well as the number of local maxima & minima must be found out which basically means extrema and its values are equal to or differ in on value only. Secondly this local minima or maximum gives the mean value which should be equal to zero. By following the above two conditions one IMF is generated & afterwards same steps are performed on the remaining residues iteratively till the monotonic function is obtained^22–24^. Figure 7 shows the flow chart for the EMD decomposition.

Consider the input signal as z (T), residual value as a (T) = z (T), number of iterations count as c = 1. The Algorithm to calculate the IMF’s can be described in the steps given below:

1. Compute all the local extremum points i.e. all the maxima and minima for given signal z (T).
2. Find the upper envelope Au (T) by joining all the local maxima with cubic spline line functions.
3. Find the lower envelope Al (T) by joining all the local minima with cubic spline line functions.
4. All the input signal data should be covered by using these Au (T) & Al (T). Then the average value of both the upper & lower envelopes helps us to calculate the mean denoted by d (T) given by
  1 $${\text{d}}\left( {\text{T}} \right) = \left[ {{\text{A}}_{{\text{u}}} \left({\text{T}} \right) + {\text{A}}_{{\text{l}}} \left( {\text{T}} \right)} \right]/{2}.$$
5. The next function s (T) can be obtained by taking the difference between d (T) mean value and z (T) input signal.
  2 $${\text{s }}\left( {\text{T}} \right) \, = {\text{ z }}\left( {\text{T}} \right) \, {-}{\text{ d }}\left( {\text{T}} \right).$$
6. This function s (T) should satisfy the two conditions given above to be an IMF. If it is satisfied, then we can find the residual value a (T).
  3 $${\text{IMF}}_{{1}} = {\text{ s }}\left( {\text{T}} \right),$$
  4 $${\text{a }}\left( {\text{T}} \right) \, = {\text{ a }}\left( {\text{T}} \right) \, {-}{\text{ s }}\left( {\text{T}} \right),$$
  5 $${\text{c }} = {\text{ c }} + { 1}.$$
  After that the next step is followed. If the function s (T) do not satisfy the above two conditions then z (T) = s (T) and duplicate all the steps from 1 to 6 again.
7. The disintegration should be stopped if we get the monotonic value of the residual function a (T). Otherwise z (T) = a (T) and again we have to repeat all the steps from 1 to 6. At the end of disintegration process whenever the constant value is obtained called as residue and further IMFs cannot be obtained finally the original signal z (T) is obtained as given in Eq. (6) below.
  6 $${\text{z }}({\textrm{T}}) =\sum_{{\text{j}}=1}^{\textrm{c}}{\text{IMF}}_{\textrm{j }}\left({\text{T}}\right)+{\textrm{a}}({\text{T}})$$

**Figure 8:**
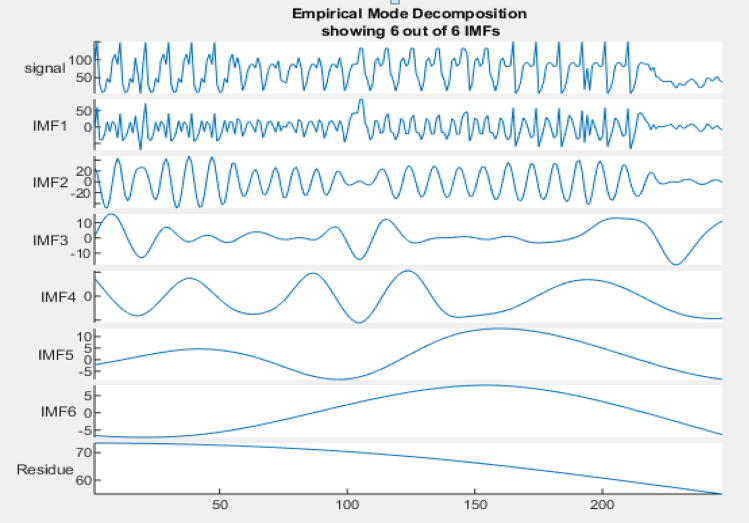
Decomposition of EEG signal by EMD into IMFs.

By following the above algorithm steps, we are able to decompose the input EEG signal value into c value of IMF & a Residue given by a (T). In proposed work six value of IMFs along with residue signal are obtained using EMD signal decomposition as shown in Fig. 8.

## Analytic signal representation of IMFs

The next step is to obtain the analytic representation of the IMF’s by Hilbert transform which is used to remove the dc offset from the signal that basically compensates the non -stationary property of the signal^16,25^. Let the real value of IMF = In (T). The Analytic representation of this real valued IMF is given in Eq. (7).

7 $${\text{x }}\left( {\text{T}} \right) \, = {\text{ In }}\left( {\text{T}} \right) \, + {\text{ I h }}\left\{ {{\text{In }}\left( {\text{T}} \right)} \right\},$$

where, h {In (T)} is Hilbert transform for In (T).

In (T) is nth IMF extracted from the signal z (T).

A linear operator is called as Hilbert transform which transforms the real valued function In (T) into another real valued function H{In (T)} with the help of convolution as given in Eq. (8).

8 $${\text{H }}\left\{ {{\text{In}}\left( {\text{T}} \right)} \right\} = {\text{ In}}\left( {\text{T}} \right)*{ 1}/\pi {\text{T}}{.}$$

Such signal can be further described in terms of instantaneous amplitude M (T) & phase φ (T) as given in Eq. (9).

9 $${\text{X }}\left( {\text{T}} \right) = {\text{ M }}\left( {\text{T}} \right){\text{ e}}^{{{\text{i}}\varphi \left( {\text{T}} \right)}} ,$$

10 $${\text{M }}\left( {\text{T}} \right) \, = {\text{In}}^{{2}} \left( {\text{T}} \right) \, + \left[ {{\text{ H}}^{{2}} \left\{ {{\text{In }}\left( {\text{T}} \right)} \right\}} \right],$$

11 $$\Phi \left( {\text{T}} \right) \, = {\text{ arctan }}\left[ {{\text{In }}\left( {\text{T}} \right)/{\text{H}}\left\{ {{\text{In }}\left( {\text{T}} \right)} \right\}} \right].$$

After performing the above operations Feature Extraction is done on these IMFs.

## Feature extraction

In this section nine statistical measures are used to get the detailed information of the EEG signal after its decomposition into six values of IMFs. The Nine features to be extracted are namely (Clf) clearance factor, (Ac) Activity, (AAC) average amplitude change, (RMS) root mean square, (ASS) absolute square root sum, (Crf) Crest factor, (Shf) Shape factor, (AS) absolute sum, (LD) log detector. Each IMF helps in generation of nine feature indexes. The generated feature matrix is of size 10 × 9 per value of data which is further given to deep learning network block. Each of the above parameter are described in detail further.

Clearance factor is the part of uppermost point of the signal to the square of average of square root of the absolute value of EEG signal. The measure of signal power is the activity of the signal. Average amplitude change is mean value of the change between two consecutive samples which characterizes liveliness of the signal. Root mean square is evaluated as the square root of the summation of the squares of each input value. Absolute square root sum is defined as summation of the square root of each sample. Crest factor shows the crests extreme present in a signal waveform. Absolute sum is defined as summation absolute values of all the input signals. The equations for above statistical features are given as below from (12)–(20):

12 $${\text{C}}_{\text{lf}} =\frac{\text{max}({y}_{i})}{{\left(\frac{1}{\text{N}}\sum_{\text{i}=1}^{\text{N}}\sqrt{\left|{\text{y}}_{\text{i}}\right|}\right)}^{2}}$$

13 $${\text{A}}_{\text{c}} ={\sigma }_{x}^{2}$$

14 $$\text{AAC }=\frac{1}{\text{N}}\sum_{\text{i}=1}^{\text{N}}\left|{\text{y}}_{\text{i}+1}- {\text{y}}_{\text{i}}\right|$$

15 $$\text{RMS }=\sqrt{\frac{1}{N} \sum_{i=1}^{N}\left({y}_{i}^{2}\right)}$$

16 $$\text{ASS }=\sum_{i=1}^{N}\sqrt{{y}_{i}}$$

17 $${\text{C}}_{\text{rf}} =\frac{\text{max}({y}_{i})}{RMS}$$

18 $${\text{S}}_{\text{hf}}=\frac{\text{RMS}}{\frac{1}{\text{N}}\sum_{\text{i}=1}^{\text{N}}\sqrt{\left|{\text{y}}_{\text{i}}\right|}}$$

19 $$\text{AS}= \sum_{i=1}^{N}\left|{y}_{i}\right|$$

20 $$\text{LD }= {e}^{\frac{1}{N}\sum_{i=1}^{N}\text{log}(\left|{y}_{i}\right|)}$$

## Deep neural network classifier

**Figure 9:**
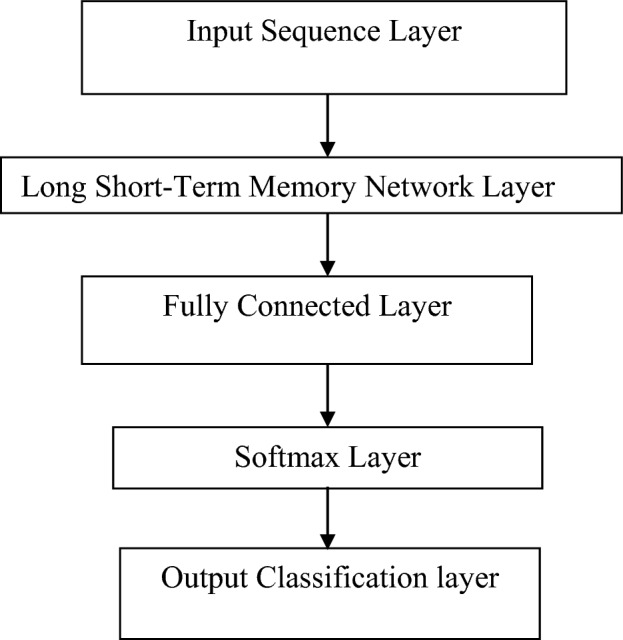
LSTM Architecture.

Machine learning algorithm are open with variety of types which is basically used for classification purpose. In the offered work deep neural Network classifier fulfil the purpose of classification. The Stacked neural network is basically called as deep neural network & as the name suggests it consists of single input& output layer with a greater number of hidden layers. Automatic Feature Extraction can be done with the help of DNN as it works on both unlabelled and unstructured data. The extracted feature as calculated from EEG signal are given as input to DNN classifier. Further the LSTM called as long short-term memory network is utilised to classify the sequence of this time series data as shown in Fig. 9.

LSTM network models also called as recurrent neural network are able to remember and learn long sequences of input data. The extracted features are given as input to first layer. The LSTM layer is the second layer that helps in label classification of the input sequence data depending on different parameters such as hidden neuron value, name & unit. The third layer is known as fully connected layer and as the name suggests it helps in connection of all inputs to each upcoming layers activation unit. In this layer multiple input is given so that each input node is multiplied with the attach weight and also bias vector addition is applied to it. Because of this there is combination of different extracted features which helps in classification of data. The size of this layer will be given by number of classes of data. The Softmax layer is the fourth layer which leads to normalization of the output according to Softmax activation function. This layer will give the positive number as output whose addition will be equal to one. The next layer is the last and fifth layer called as classification layer that will consider the probability value obtained from the previous layer which ultimately leads to classification of input data. The diagram in Fig. 9 shows the LSTM Architecture using DNN.

The building blocks of an LSTM-based deep neural network (DNN) classifier are input layers that receive sequential data, LSTM layers that capture temporal dependencies, and hidden layers that may be added for additional feature extraction. Final predictions are produced by the output layer, and parameters are optimized during training by minimizing a selected loss function and via backpropagation. It is essential to adjust hyper parameters such as the number of LSTM layers, units per layer, and dropout rate. LSTM-based DNN classifiers are useful for a variety of sequential data tasks of time series data because performance of evaluation using validation data comes before testing on unseen data.

A key component of deep neural network (DNN) classifiers, the Long Short-Term Memory (LSTM) technique uses memory cells and gates to control input flow. These gates regulate memory states by keeping or deleting information on a per-selective basis. They include forget, input, and output gates. Long-range relationships in sequential data are efficiently captured by LSTMs through forget gates, which determine the significance of previous data, input gates, which assess fresh input, and output gates, which control data flow. LSTMs perform well in tasks like classification, prediction, and generation because backpropagation is used to optimize parameters during training.

## Results

### Evaluation metrics

In the Proposed work 4 normal person & 4 neurological disorder patient data was used for analysis purpose. Nine Parameters are used to assess the DNN Classifier Performance as described in the above section with the help of feature matrix. fourfold cross validation are useful to assess the performance.

**Figure 10:**
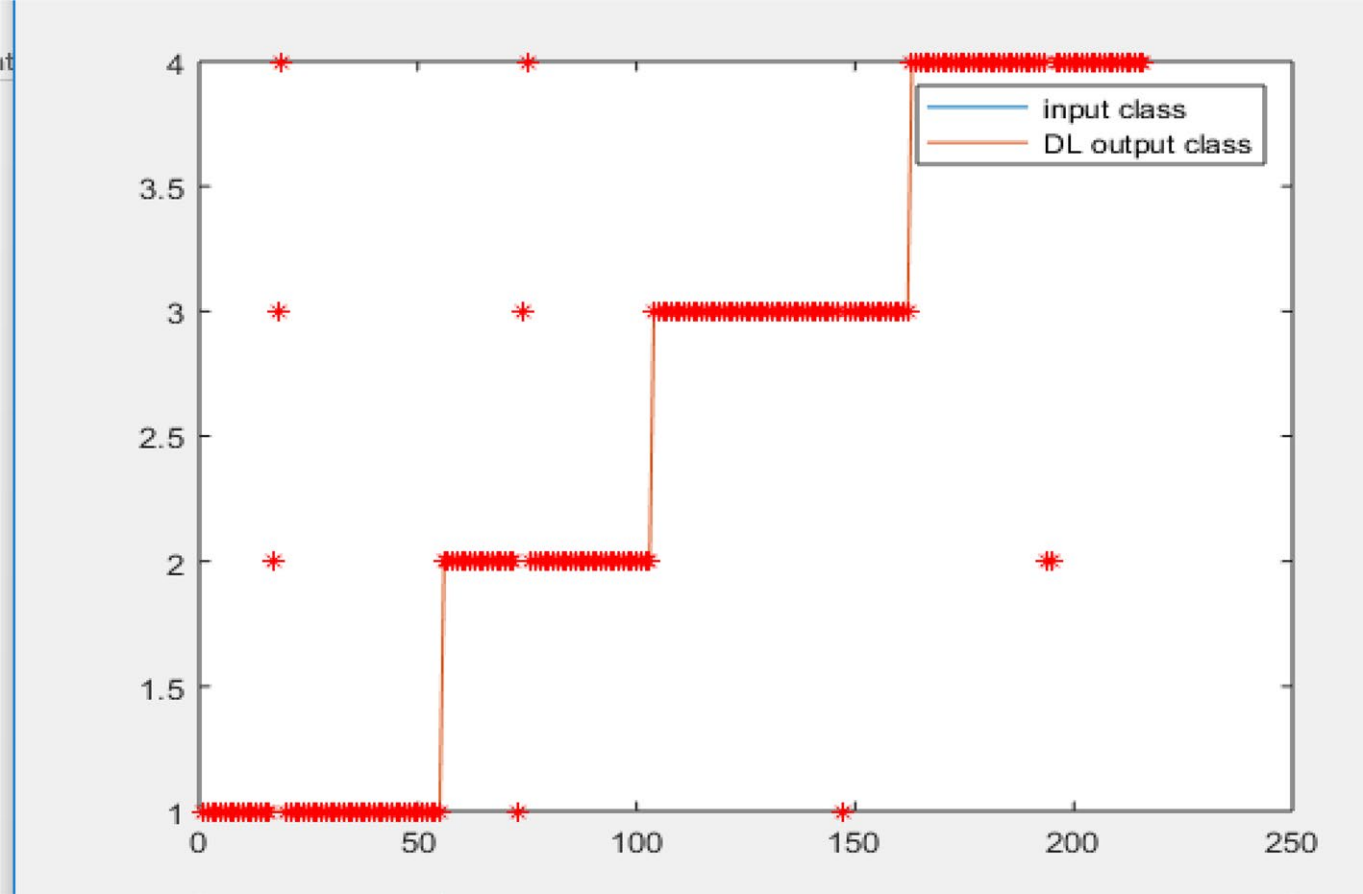
Classification using DNN for 216 samples with Accuracy = 97%.

In all 80% data as captured by the hardware is used for the training while 20% is utilized for testing. Figure 10 shows Classification using DNN for 216 samples with Accuracy = 97% for acquired database from the hardware.

**Figure 11:**
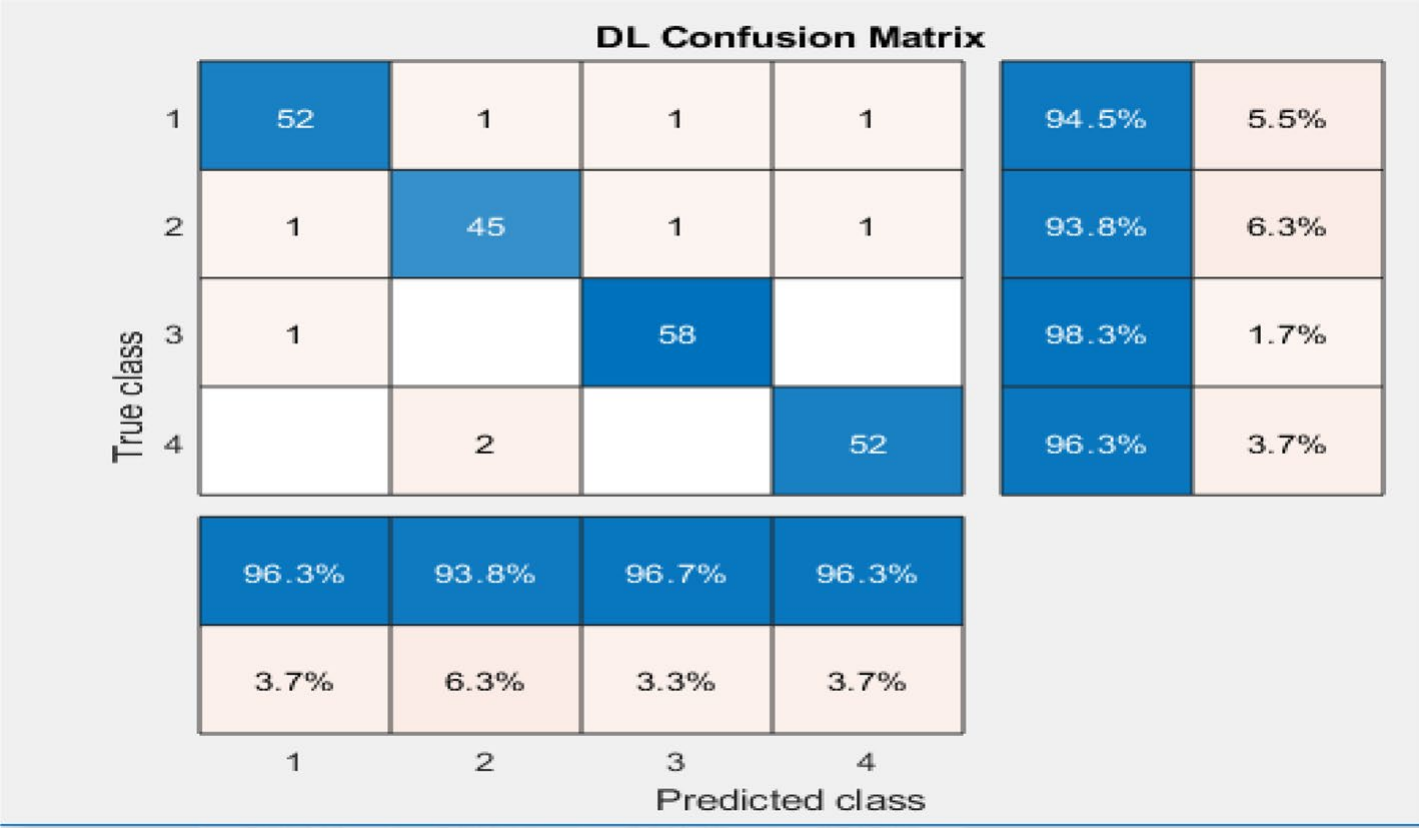
Predicted Class vs True class confusion matrix.

Table 2. Shows the time frequency representation value of the confusion matrices. Further Evaluation Metrics are calculated from the values given in table along with various parameters like Sensitivity, accuracy, F1-Score, precision and specificity. Figure 11 depicts the Confusion Matrix for predicted Class vs. True class.

**Table 2:** True class vs. Predicted class Confusion matrix for DNN classification.

Actions	Deep Neural network classification with EMD decomposition			
	Class 1	Class 2	Class 3	Class 4
True positive (TP)	52	45	58	52
False positive (FP)	2	3	2	2
False Negative (FN)	3	3	1	2
True Negative (TN)	159	165	155	160

Class 1 has the highest number of TP (52) and relatively low FP (2), indicating good performance in correctly identifying instances of this class. Class 3 has a high FN (1) compared to other classes, suggesting some instances belonging to Class 3 were incorrectly classified as other classes. The overall performance can be further analyzed using additional metrics like accuracy, precision, recall, specificity, and F1 score to provide a comprehensive evaluation of the classifier's performance.

### Performance parameter

The various performance parameters to be calculated are defined as below^3^

21 $${\text{Accuracy}}\left( \% \right) \, = \,\frac{{{\text{TP}} + {\text{TN}}}}{{{\text{TP}} + {\text{FP}} + {\text{FN}} + {\text{TN}}}} \times 100.$$

22 $${\text{Precision}}\left( \% \right) \, = \frac{{{\text{TP}}}}{{{\text{TP}} + {\text{FP}}}} \times { 1}00.$$

23 $${\text{Sensitivity}}\left( \% \right) \, = \frac{{{\text{TP}} }}{{{\text{TP}} + {\text{FN}}}} \times { 1}00.$$

24 $${\text{Specificity}}\left( \% \right) \, = \,\frac{{{\text{TN}}}}{{{\text{TN}} + {\text{FP}}}} \times 100.$$

25 $${\text{F1}} - {\text{Score }} = \,\frac{{{2 }*{\text{ precision }} \times {\text{ Recall}}}}{{{\text{Precision }} + {\text{ Recall}}}}.$$

where, $${\text{Precision}}\left( \% \right) \, = \frac{{{\text{TP}}}}{{{\text{TP}} + {\text{FP}}}},$$$${\text{Recall }} = \,\frac{{{\text{TP}}}}{{{\text{TP}} + {\text{FN}}}}.$$

**Figure 12:**
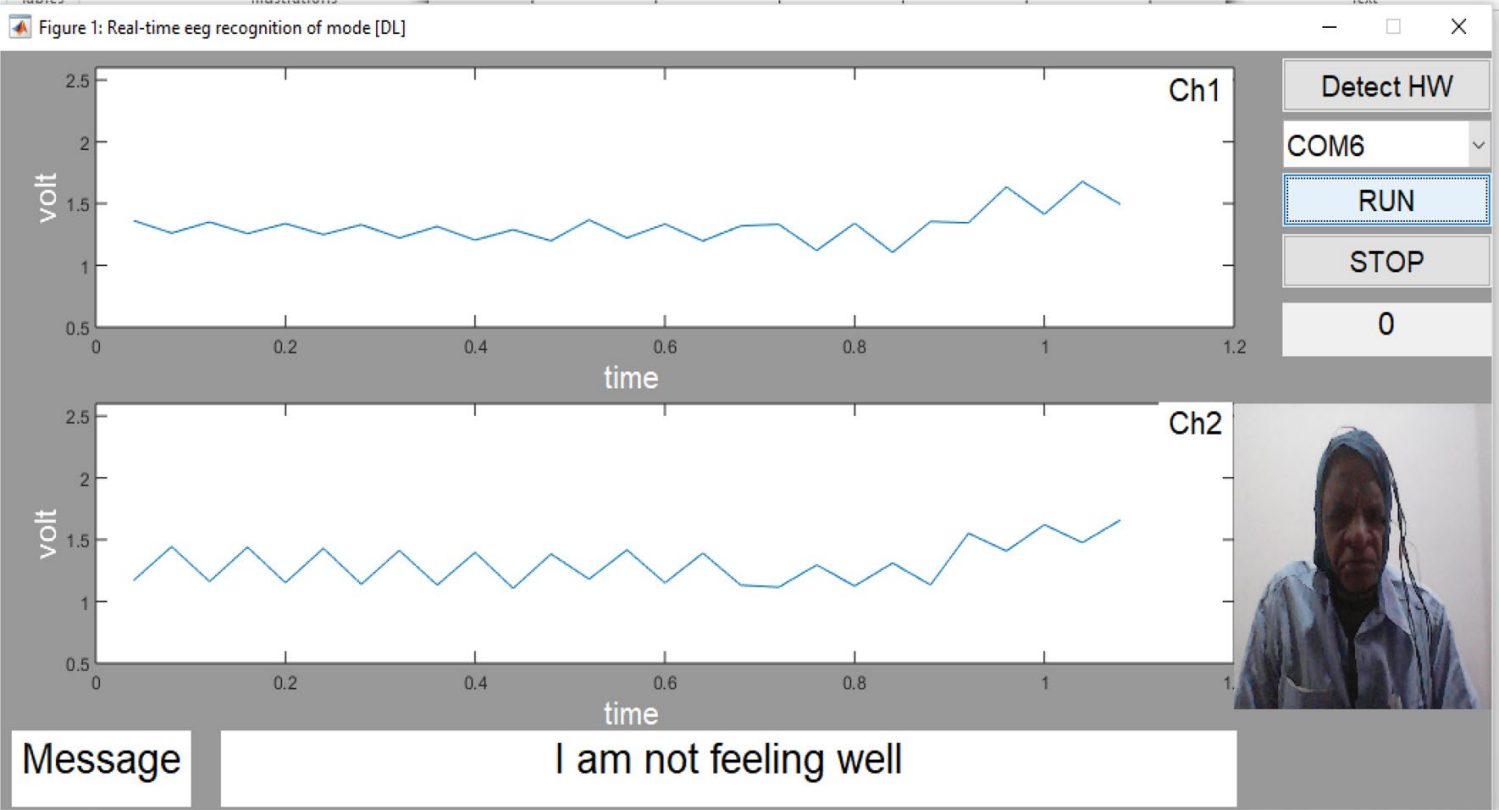
Real time Testing of the message “I am not feeling well” by using Deep learning.

**Figure 13:**
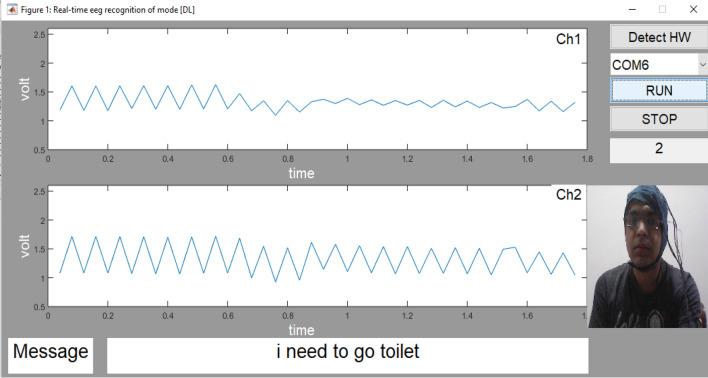
Real time Testing of the message “I need to go toilet” by using Deep learning.

Various performance parameters are calculated with the help of the above five equations viz, ((21), (22), (23), (24), (25)) as shown in the Table 3. In the proposed work in real time analysis was also done to evaluate the performance. In real time the performance accuracy of about 85% is obtained for the four messages as shown in the Table 4. The tested output message ‘I am not feeling well’ of subject 1 as displayed in the output is shown in Fig. 12. The tested output message ‘I need to go to toilet’ of subject 2 as displayed in the output is shown in Fig. 13.

**Table 3:** Performance Parameter.

Performance parameter	Class 1	Class 2	Class 3	Class 4
Accuracy (%)	97	97	98	98
Precision (%)	96	94	97	96
Sensitivity (%)	95	94	98	96
Specificity (%)	99	98	99	99
F1 score	0.98	0.96	0.98	0.98

**Table 4:** Percentage accuracy for messages in real time by using DNN Classifier.

Sr. no	Subject no	Communication message	No of times experimentation done (A)	Time required to response	No of samples correctly Identified (B)	Percentage accuracy (A*100/B)
1	Subject1	I need Food	40	10 s	32	80
		I need a glass of water	40	10 s	35	87.5
		I need to go toilet	40	10 s	36	90
		I am not feeling well	40	10 s	36	90
2	Subject2	I need Food	40	10 s	33	82.5
		I need a glass of water	40	10 s	36	90
		I need to go toilet	40	10 s	37	92.5
		I am not feeling well	40	10 s	32	80
3	Subject3	I need Food	40	10 s	31	77.5
		I need a glass of water	40	10 s	34	85
		I need to go toilet	40	10 s	35	87.5
		I am not feeling well	40	10 s	35	87.5
4	Subject4	I need Food	40	10 s	33	82.5
		I need a glass of water	40	10 s	34	85
		I need to go toilet	40	10 s	36	90
		I am not feeling well	40	10 s	30	75
5	Subject5	I need Food	40	10 s	31	77.5
		I need a glass of water	40	10 s	32	80
		I need to go toilet	40	10 s	34	85
		I am not feeling well	40	10 s	31	77.5
6	Subject6	I need Food	40	10 s	31	77.5
		I need a glass of water	40	10 s	33	82.5
		I need to go toilet	40	10 s	33	82.5
		I am not feeling well	40	10 s	33	82.5
7	Subject7	I need Food	40	10 s	29	72.5
		I need a glass of water	40	10 s	34	85
		I need to go toilet	40	10 s	34	85
		I am not feeling well	40	10 s	32	80
		I need a glass of water	40	10 s	34	85

The Work contributes to the benefits the society as it helps parlayed person to improve quality of life by enabling communication between patient and external world. The system solves the speech impairment problem of paralyzed person, aphasia, cerebral palsy and Parkinson’s diseases which is need of the society. Society can use the communication device is available at low cost. A cheap and efficient system for real time communication by using only EEG Signal. After acquiring data from the proposed hardware, various soft computing methods are deployed after feature extraction and classification which makes the hardware robust. The Device the help the people from medical fraternity, people across the globe suffering from this disorder. The Work focuses on improving the quality of life of disabled person by enabling communication between the patient and the external world (family members, Caretakers, Doctors) by acquiring bio potential signals like EEG and by decomposing the signal, feature extraction and applying machine learning algorithm so as to classify the brain states or gestures which will be useful to communicate different basic and needy messages by the patients viz, I Need a glass of water, I need food, I am feeling uneasy, I want to go to toilet etc.

## Conclusion

In this paper, we assess the performance of DNN based on EEG signal classification both with the help of our acquired database from developed hardware as well as in real time. Here feature was extracted by EMD decomposition which ultimately leads to classification of the data into four classes. This further leads to depiction of brain states via four different messages from neurological disorder patient. The results are successfully authenticated using DNN and provide average accuracy of about 97%. In real time processing Classification accuracy obtained is 85% which outperforms other available methods as until now none of the work was done on the personalised database & in real time.The significance and advantage of the proposed method are Novelty, Personalized Database, Real-Time Processing, High Classification Accuracy, Outperformance in Real-Time Processing. Compared to generic databases, the database used in the study was obtained via developed hardware, which probably captures more precise and pertinent EEG signals for the intended use. The results' relevance and applicability are improved by this customized method. More resilient and discriminating characteristics for classification may result from this strategy. The study asserts that the application of real-time processing and the use of a personalized database are both innovative. Through the development of these methods, the work advances the field of EEG data processing and creates new opportunities for real-time, individualized neurological applications.

## Limitations and future scope

The limitations of the proposed work are Computational Complexity, Noise Susceptibility and Generalizability across Datasets. EEG hardware may face limitations in processing power, hindering real-time analysis. Complex algorithms could overwhelm the hardware, leading to latency or reduced performance. EEG signals are prone to various sources of noise, impacting signal quality. Hardware limitations, such as inadequate shielding or electrode quality, can exacerbate this issue, affecting measurement accuracy. Variability in EEG hardware specifications, including electrode configuration and sampling rates, can hinder generalization across different datasets. This inconsistency poses challenges in developing universally applicable signal processing methods.

The future scope of the proposed work includes Dataset Validation, Real-time Optimization, Robustness Testing, Feature Selection and Fusion, User Interface Design**,** Interpretability Features**,** Clinical Validation and Feedback. Validation of the proposed method with larger and more diverse datasets to ensure its generalizability across different patient populations and EEG recording conditions. Optimization of the real-time implementation of the method for computational efficiency by considering techniques such as model compression, hardware acceleration, or algorithmic optimizations to reduce computational burden and latency. Performance of robustness testing to evaluate the method's performance against various real-world factors, including noise, artifacts, electrode placement variability, and patient-specific characteristics. Exploration of different feature selection techniques to identify the most informative features for classification. Investigate feature fusion strategies to combine information from multiple sources, such as different IMFs or other physiological signals, to improve classification performance. Design of an instinctive and user-friendly edge for patients to interact with system, incorporating visualizations or feedback mechanisms to enhance understanding of brain activity and improve user engagement. In Future Provide interpretable insights into the classification results to enhance user trust and acceptance. Incorporate features that allow users to interpret the system's outputs and understand the basis for classification decisions. Collaborate with clinicians and end-users to validate the effectiveness and usability of the system in real-world clinical settings. Solicit feedback from patients and healthcare professionals to refine the system design and address specific user needs and preferences.

## Data Availability

The datasets used during the current study are available from the corresponding author on reasonable request.
